# Persistent accumulation of unrepaired DNA damage in rat cortical neurons: nuclear organization and ChIP-seq analysis of damaged DNA

**DOI:** 10.1186/s40478-018-0573-6

**Published:** 2018-07-26

**Authors:** Jorge Mata-Garrido, Olga Tapia, Iñigo Casafont, Maria T. Berciano, Ana Cuadrado, Miguel Lafarga

**Affiliations:** 1 Department of Anatomy and Cell Biology and “Centro de Investigación Biomédica en Red sobre Enfermedades Neurodegenerativas” (CIBERNED), University of Cantabria-IDIVAL, Santander, Spain; 2 Chromosome Dynamics Group, Molecular Oncology Program, Spanish National Cancer Research Centre (CNIO), 28029 Madrid, Spain; 3 Present address: Department of Molecular Biology and CIBERNED, University of Cantabria-IDIVAL, Santander, Spain; 4 Department of Anatomy and Cell Biology, Faculty of Medicine, Avd,. Cardenal Herrera Oria sn, /39011 Santander, Spain

**Keywords:** DNA damage- ionizing radiation- cortical neurons- persistent DNA damage foci- transcription silencing- CTCF- γH2AX genomic distribution, Neurodegenerative diseases

## Abstract

Neurons are highly vulnerable to DNA damage induced by genotoxic agents such as topoisomerase activity, oxidative stress, ionizing radiation (IR) and chemotherapeutic drugs. To avert the detrimental effects of DNA lesions in genome stability, transcription and apoptosis, neurons activate robust DNA repair mechanisms. However, defective DNA repair with accumulation of unrepaired DNA are at the basis of brain ageing and several neurodegenerative diseases. Understanding the mechanisms by which neurons tolerate DNA damage accumulation as well as defining the genomic regions that are more vulnerable to DNA damage or refractory to DNA repair and therefore constitute potential targets in neurodegenerative diseases are essential issues in the field. In this work we investigated the nuclear topography and organization together with the genome-wide distribution of unrepaired DNA in rat cortical neurons 15 days upon IR. About 5% of non-irradiated and 55% of irradiated cells accumulate unrepaired DNA within persistent DNA damage foci (PDDF) of chromatin. These PDDF are featured by persistent activation of DNA damage/repair signaling, lack of transcription and localization in repressive nuclear microenvironments. Interestingly, the chromatin insulator CTCF is concentrated at the PDDF boundaries, likely contributing to isolate unrepaired DNA from intact transcriptionally active chromatin. By confining damaged DNA, PDDF would help preserving genomic integrity and preventing the production of aberrant proteins encoded by damaged genes.

ChIP-seq analysis of genome-wide γH2AX distribution revealed a number of genomic regions enriched in γH2AX signal in IR-treated cortical neurons. Some of these regions are in close proximity to genes encoding essential proteins for neuronal functions and human neurodegenerative disorders such as *epm2a* (Lafora disease), *serpini1* (familial encephalopathy with neuroserpin inclusion bodies) and *il1rpl1* (mental retardation, X-linked 21). Persistent γH2AX signal close to those regions suggests that nearby genes could be either more vulnerable to DNA damage or more refractory to DNA repair.

**Electronic supplementary material:**

The online version of this article (10.1186/s40478-018-0573-6) contains supplementary material, which is available to authorized users.

## Introduction

Neuronal DNA damage with generation of double strand breaks (DSBs) occurs physiologically as a result of transcription by means of the activity of topoisomerase complexes, which cut transiently both DNA strands to release torsional stress. [13, 37, 47, 72, 74]. As a consequence, repair of such topoisomerase II-induced DNA damage represents an endogenous threat for gene expression and may lead to unrepaired DNA accumulation and generation of transcriptional errors potentially harmful for the cell [26, 27]. An additional source of endogenous neuronal DNA damage is the oxidative stress produced by the high rate of oxygen consumption, which leads to increased generation of reactive oxygen species with age [73]. Mammalian neurons are also highly vulnerable to exogenous genotoxic agents such as chemotherapy drugs and ionizing radiations (IRs) normally used in cancer treatment [7, 50]. Such vulnerability to DNA damage is in part mediated by the relaxed chromatin conformation characteristic in neurons, that facilitates the access of genotoxic agents to DNA strands [50, 53].

Unrepaired DNA accumulation results in loss of genome integrity and the subsequent increased risk of errors in the manufacture of both RNA and protein products [13, 27]. Such increase of unrepaired DNA lesions might contribute to the ageing process: for instance, an age-dependent decline in DNA repair activity has been observed in the rodent and human brain, and defects in DNA repair may cause premature aging [23, 43, 45, 73, 79]. Persistent accumulation of DNA damage has also been linked to several neurodegenerative diseases both in human patients and experimental animal models [3, 36, 48, 52, 59, 67]. In fact, oxidative DNA damage is emerging as a hallmark in Alzheimer’s and Parkinson’s diseases [44, 69].

DSBs are detrimental for neurons as they profoundly impact on genome integrity, transcriptional activity, cellular proteostasis and energy starvation [7, 21, 27, 48, 50, 59]. They are repaired by non-homologous end joining (NHEJ) since in post-mitotic neurons there is not a normal sister chromatid sequence that serves as a template. NHEJ is more error-prone than homologous recombination and small deletions can be introduced during the DSB ends processing before religation [13, 41].

In a previous study, using a model to induce DSBs in peripheral nervous system neurons of the rat sensory ganglia by means of IR with X-rays (4 Gy), we demonstrated that the neuronal DNA damage response (DDR) involves the formation of two types of DNA-damage/repair chromatin domains [7]. The first type is featured by transient and abundant small foci that disappear within the 24 h post-IR, reflecting a rapid and effective DNA repair crucial for neuronal survival. The second consists of one to three large and persistent DNA damage foci (PDDF) that accumulate unrepaired DNA for several weeks post-IR [7]. Importantly, PDDF preserve DNA damage signaling and repair factors, are transcriptionally silent and associate with repressive nuclear environments.

To expand on the aforementioned study in peripheral nervous system neurons, we aimed to determine whether the accumulation of unrepaired DNA in PDDF is a general cellular event that also affects neurons of the central nervous system, in particular, cerebral cortex neurons. With this purpose we investigated the nuclear organization and fate of unrepaired DNA accumulated in PDDF of cortical neurons 15 days upon irradiation (4 Gy). We also wanted to define the identity of the genomic sequences enriched within PDDF. With this purpose, we used ChIP-seq to obtain the genome-wide distribution of γH2AX, which specifically recognizes the damaged DNA accumulated in these neuronal foci [7, 50]. Our results show that the DDR pattern observed in cortical neurons reproduces the structural, spatial, molecular and transcriptional organization of the PDDF present in peripheral nervous system neurons, suggesting a common long-term response of mammalian neurons to DSB lesions. Moreover, ChIP-seq analysis of damaged DNA in PDDF identifies a number of genomic regions enriched in γH2AX signal, some of them in close proximity to genes encoding essential proteins for neuronal functions and neurodegenerative disorders. Importantly, since the expression of some of these genes appears affected both in control and, to a higher extent, in irradiated neurons, our ChIP-seq has allowed to identify genes either more vulnerable to DNA damage or more refractory to DNA repair, which are potential targets in neurodegenerative diseases.

## Materials and methods

### Animals

Experiments were designed and performed to minimize the use of animals. A total of 42 young male Sprague–Dawley rats, distributed in a control (non-irradiated, *n* = 21) and X-ray irradiated animals (*n* = 21), and 6 young male C57 mice (non-irradiated, *n* = 3; irradiated, *n* = 3) were used. Irradiated animals received a single dose of 4 Gy of ionizing radiation (IR). Animals were housed with a 12 h light/dark cycle and had free access to food and water. They were kept, handled, and sacrificed according with the directives of the Council of the European Communities and current Spanish legislation, and the experiments were approved by the Bioethical Committee of the University of Cantabria.

### X-ray irradiation

Exogenous DNA damage was induced by X-Ray irradiation using an X-Ray generator system (Maxishot-d, Yxlon, Int. USA) equipped with an X-Ray tube that works at 200 kV and 4.5 mA. The animals, deeply anesthetized with pentobarbital (50 mg/kg), were protected with a lead tube, exposing only the head, and the beam focused on the head to avoid adverse effects produced by global animal radiation. Animals received a single sub-lethal dose of 4Gy, a reference dose in DNA damage/repair experiments [7, 50]. Control and irradiated animals were sacrificed 15 days (15d) post-IR and the cerebral cortex was isolated and processed for different cell biology and biochemical procedures.

### Immunofluorescence and confocal microscopy

For light immunocytochemistry, animals (*n* = 3 per experimental condition) deeply anesthetized as described above were perfused with the fixative solution containing 3.7% formaldehyde (freshly prepared from paraformaldehyde) in PBS. Tissue fragments from the parietal cortex were removed and washed in PBS. Each tissue fragment was transferred to a drop of PBS on a siliconized slide (SuperFrostPlus, Menzel-Gläser, Germany) and squashed preparations of dissociated neurons were performed following the previously reported procedure [61]. In addition to dissociated neuron preparations we performed 7 μm-thick formaldehyde-fixed cryosections from the parietal cortex. All samples were sequentially treated with 0.1 M glycine in PBS for 15 min, 3% BSA in PBS for 30 min and 0.5% Triton X-100 in PBS for 45 min. They were then incubated with primary antibodies overnight at 4 °C, washed with 0.05% Tween 20 in PBS, incubated for 45 min in the specific secondary antibody conjugated with FITC or Cy3 (Jackson, USA), washed in PBS and mounted with the antifading medium ProLong (Thermo Fisher Scientific). Some samples were counterstained with Propidium Iodide (Thermo Fisher Scientific), a cytochemical marker of nucleic acids.

Confocal images were obtained with a LSM510 (Zeiss, Germany) laser scanning microscope using a 63× oil (1.4 NA) objective. To avoid overlapping signals, images were obtained by sequential excitation at 488 and 543 nm in order to detect FITC and Cy3, respectively. Images were processed using Photoshop software.

The quantitative analysis of the i) proportion of damaged cortical neurons containing IR-induced PDDF, ii) mean number of foci per nucleus within the population of PDDF-containing neurons, and iii) nuclear topography of PDDF in three nuclear regions (perinucleolar, adjacent to heterochromatin clumps and nuclear interior) was performed in dissociated rat cortical neurons. Samples were immunostained for γH2AX, counterstained with propidium iodide and directly examined throughout different focal planes using a 40× objective and fluorescence microscopy (Axioskop 2 plus, Zeiss, Germany). At least 100 neurons per animal were examined (*n* = 3 animals per experimental condition). Image processing and measurement steps were performed on ImageJ, public domain software for image analysis (NIH, Bethesda, Maryland, USA; http://rsb.info.nih.gov/ij/). Average values were pooled for subsequent graphing and analysis. Data were analyzed using Microsoft Excel and the analysis of variance was used to determine the statistical significance of differences between control and irradiated neurons of sensory ganglia. Values are Means SD.

### Transmission and immunoelectron microscopy

For conventional and immunogold electron microscopy examination of cortical neurons, control and irradiated rats (*n* = 3 animals per group) were perfused under deep anesthesia with 3.7% paraformaldehyde in 0.1 M cacodylate buffer for 10 min at room temperature. Small tissue fragments from the parietal cortex were washed in 0.1 M cacodylate buffer, dehydrated in increasing concentrations of methanol at − 20 °C, embedded in Lowicryl K4 M at − 20 °C and polymerized with ultraviolet irradiation. Ultrathin sections were mounted on nickel grids, stained with lead citrate and uranyl acetate and examined with a JEOL 1011 electron microscope. For immunogold electron microscopy, sections were sequentially incubated with 0.1 M glycine in PBS for 15 min, 5% BSA in PBS for 30 min and the primary antibody for 2 h at 37 °C. After washing, sections were incubated with the specific secondary antibodies coupled to 10 nm gold particles (BioCell, UK; diluted 1:50 in PBS containing 1% BSA). Following immunogold labeling, grids were stained with lead citrate and uranyl acetate. As controls, ultrathin sections were treated as described omitting the primary antibody.

### In situ run-on transcription assay

Active transcription sites were labeled by incorporation of 5′-fluorouridine (5’-FU) into nascent RNA. Briefly, anesthetized control and irradiated rats (*n* = 3 animals per group) were given an intravenous injection of 5’-FU (Sigma, UK) from a stock solution of 0.4 M 5’-FU in 0.9% saline at 5 μl/g doses. All animals were sacrificed at 45 min post-injection and fixed by perfusion with 3.7% paraformaldehyde in HPEM buffer (2× HPEM: Hepes, 60 mM; Pipes, 130 mM; EGTA, 20 mM; and MgCl_2_·6H_2_O, 4 mM) containing 0.5% Triton X-100 for 10 min. Tissue samples from the parietal cortex were removed, washed in HPEM buffer containing 0.5% Triton X-100 for 10 min, dehydrated in increasing concentrations of methanol at − 20 °C, embedded in Lowicryl K4M at − 20 °C and polymerized with ultraviolet irradiation. Ultrathin sections were mounted on nickel grids and sequentially incubated with 0.1 M glycine in PBS for 15 min, 5% BSA in PBS for 30 min and the mouse monoclonal anti-BrdU (clone BU-33, Sigma, UK) antibody (diluted 1/25 in 50 mM Tris–HCl, pH 7.6, containing 1% BSA and 0.1 M glycine) for 1 h at 37 °C. After washing, sections were incubated with an anti-mouse secondary antibody coupled to 15 nm gold particles (BioCell, UK; diluted 1:50 in PBS containing 1% BSA). Following immunogold labeling, grids were stained with lead citrate and uranyl acetate and examined with a JEOL 1011 electron microscope. As controls, ultrathin sections were treated as described in absence of the primary antibody.

### SDS-PAGE and immunoblotting

Tissue samples of the parietal cortex from control and irradiated rats (*n* = 3 animals per group) were lysed using a Polytron PT-2000 (Kinematica®, Luzern-Switzerland) on ice in cold extraction buffer NETN [20 mM Tris-HCl pH 8.0, 500 mM NaCl, 1 mM EDTA] containing Benzonase (1 μL/mL lysis buffer) (Novagen), supplemented with protease and phosphatase inhibitor cocktail (Halt™ Protease and Phosphatase inhibitor single use cocktail, Thermo Scientific, USA) and incubated for 30 min on ice. After centrifugation (12 min at 12000 rpm) at 4 °C the supernatant was frozen. Proteins were separated on SDS-PAGE gels and transferred to nitrocellulose membranes by standard procedures. Protein bands were detected with an Odyssey™ Infrared-Imaging System (Li-Cor Biosciences) according to Odyssey™ Western-Blotting Protocol. Immunoblots were developed with anti-mouse IRDye800DX or anti-rabbit IRDye700DX (Rockland Immunochemicals, USA) secondary antibodies.

### ChIP-seq and ChIP-qPCR analysis

Chromatin immunoprecipitation (ChIP) of the parietal cortex neurons from control and irradiated rats (*n* = 2 animals per group) was performed as described [16], with some modifications. 20 million cells per condition were cross-linked with 1% formaldehyde added to the media for 15 min at RT. After quenching with 0.125 M Glycine, fixed cells were washed twice with PBS containing 1 μM PMSF and protease inhibitors, pelleted and lysed in lysis buffer (1%SDS, 10 mM EDTA, 50 mM Tris-HCl pH 8.1) at 2 × 10^7^ cells/ml. Sonication was performed with a Covaris system (shearing time 20 min, 20% duty cycle, intensity 6, 200 cycles per burst and 30 s per cycle). 10^7^ cells equivalent to 40–50 μg of chromatin were used per immunoprecipitation reaction with 10 μg of anti-histone H2AX phospho-Ser139 or anti-CTCF (07–729, Millipore). For ChIP-seq, 5 ng of immunoprecipitated chromatin (as quantitated by fluorometry) were electrophoresed on an agarose gel and independent sample-specific fractions of 100–200 bp were taken. Adapter-ligated library was completed by limited-cycle PCR with Illumina PE primers (11 to 13 cycles). DNA libraries were applied to an Illumina flow cell for cluster generation and sequenced on the Illumina Genome Analyzer IIx (GAIIx). Image analysis was performed with Illumina Real Time Analysis software (RTA1.8).

Alignment of 40-bp long sequences to the reference genome (RGSC6.0/rn6 rat genome) was performed using Bowtie1 (http://bowtie-bio.sourceforge.net/index.shtml) under default settings. Duplicates were removed using Picardtools (version 1.60) and peak calling was carried out using MACS2 (version 2.1.1.20160309) setting a q value (FDR) to 0.05 using the ‘--extsize’ argument with the values obtained in the ‘macs2 predictd’ step. Sample C1 was used as control for both conditions I1 and I2.

Mean read density profiles and read density heatmaps for different chromatin binding proteins were generated with deepTools 2.0 [65] using BAM files of processed reads and plotting them around peak summits called in control condition.

ChIP-qPCR on immunoprecipitated chromatin was performed using the SYBR Green PCR Master Mix and an ABI Prism® 7900HT instrument (Applied Biosystems®). Primers were designed using OligoPerfect Designer™ (Invitrogen) and reactions were performed in triplicate. Chromosome coordinates of the positions in the study and the corresponding primers are listed in Additional file 1: Table S1. The relative amount of each amplified fragment was estimated with respect to the amplification obtained from input DNA, and normalized against the binding to a negative region in the “Control 1” condition using the ΔΔCt method.

### Antibodies

The following primary antibodies were used. Mouse monoclonal antibodies anti-histone H2AX phospho-Ser139 (1/1000 Western, 1/200 immunostaining; Millipore-Upstate 05–636, MA, USA), anti-BrdU (1/25 immunostaining; Sigma B8434, UK), anti-WRAP53 (1/200 immunostaining; Abnova H00055135-M04, USA), anti-UBF (1/100 immunostaining; Santa Cruz Biotechnology SC-13125) and anti-B23 (1/100 immunostaining; Abcam ab10530). Rabbit polyclonal antibodies anti-histone H2AX phospho-Ser139 (1/200 immunostaining; Novus Biologicals NB100–384), anti-trimethyl-histone H4 (Lys 20) (1/250 immunostaining; Millipore-Upstate 07–463, USA), anti-53BP1 (1/100 immunostaining; Bethyl Laboratories A300-272A, Inc., USA), anti-CTCF (1/100 immunostaining; Millipore-Upstate 07–729, MA, USA), anti- Iba1 (1/500 immunostaining; Wako 019–19,741), anti-GFAP (1/500 immunostaining; Thermo Fisher Scientific PA3–16727), anti-NeuN (1/100 immunostaining; Abcam ab177487) and anti-Histone H3 (1/2000 Western; Thermo Fisher Scientific PA5–16183). Chicken polyclonal anti-β-galactosidase (1/200 immunostaining; Abcam ab9361). Specific secondary antibody conjugated with FITC or TexasRed were used (Jackson Lab., USA).

## Results

### Organization of PDDF induced by IR in cortical neurons

**Fig. 1:**
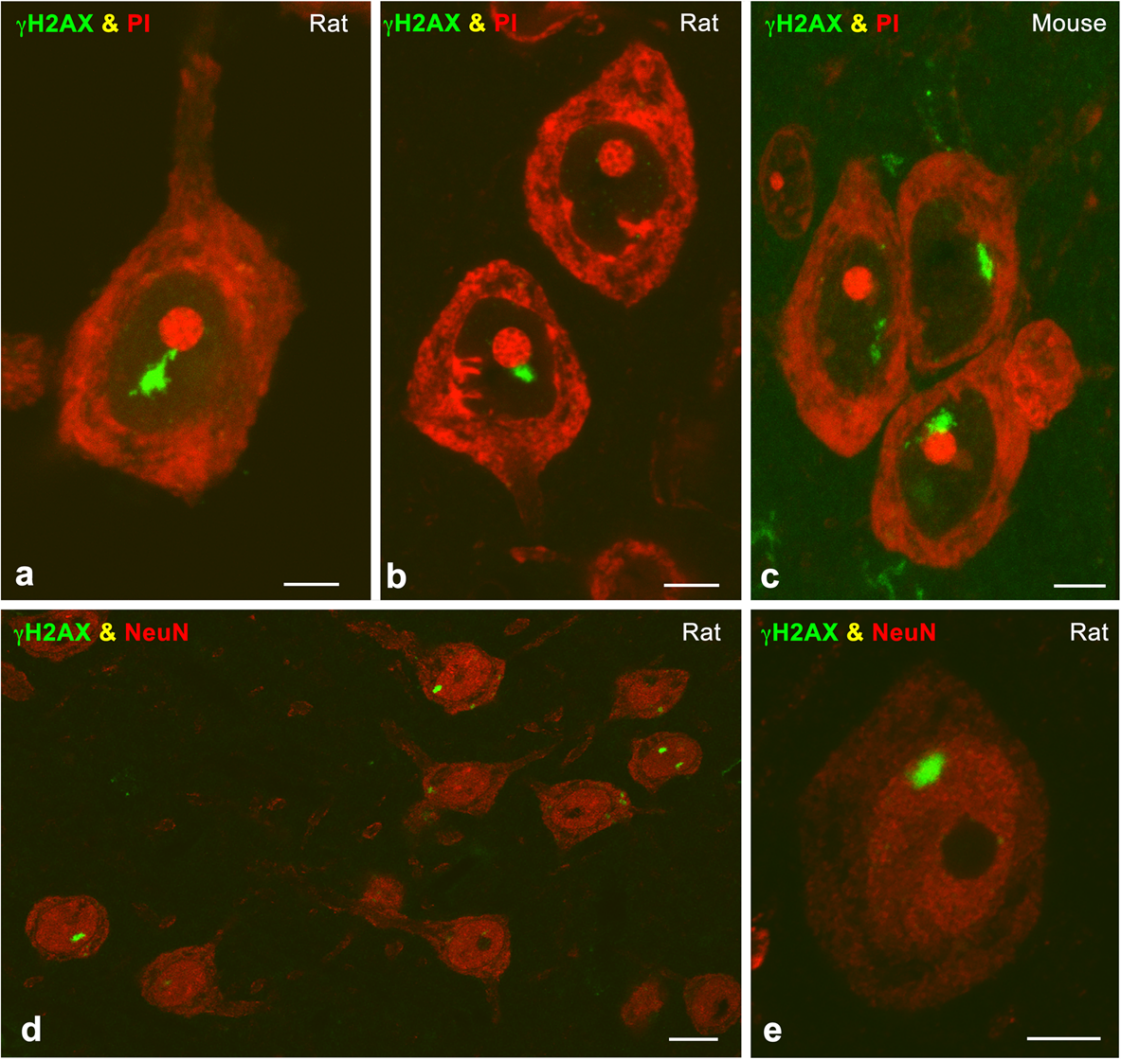
**a-c** Representative examples of immunolabeling for γH2AX of cortical neurons in a dissociated neuron preparation (**a**) and cryosections (**b**, **c**) counterstained with propidium iodide (PI) from irradiated rat (**a**, **b**) and mouse (**c**) at 15 days post-IR. Note the presence of typical PDDF associated with the nucleolus. **d, e** Rat cerebral cortex cryosections double immunolabeled for γH2AX and NeuN illustrate the specific localization of PDDF in NeuN-positive neurons at 15 days post-IR. Scale bars: a-c, e, 5 μm; d, 10 μm

**Fig. 2:**
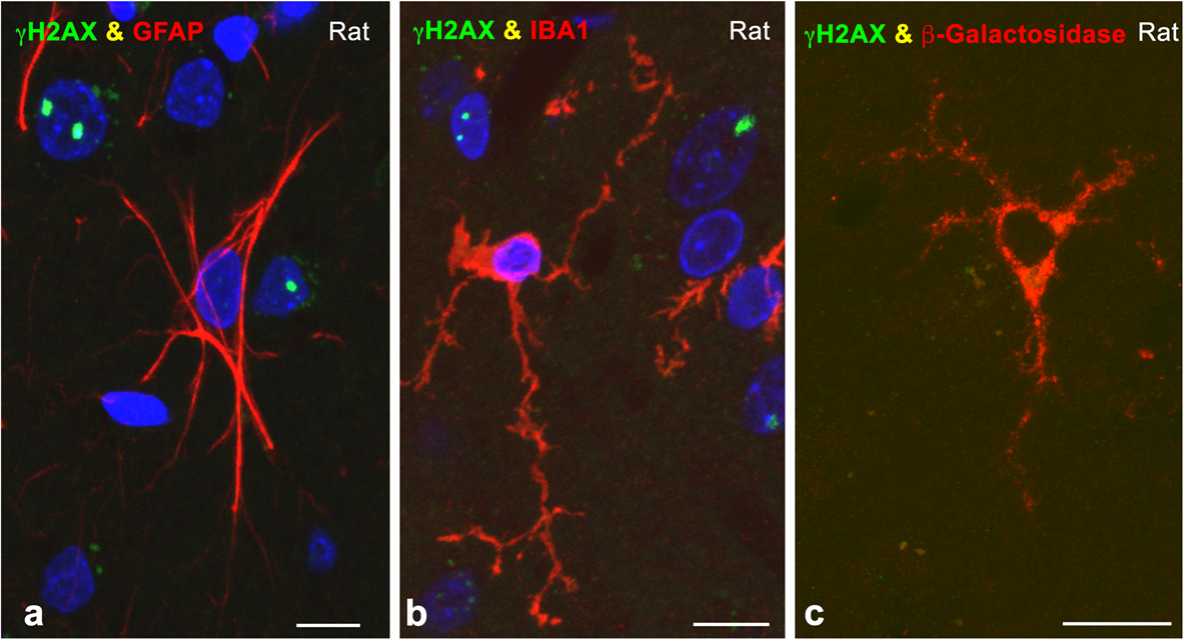
**a, b** Cerebral cortex cryosections double immunolabeled for γH2AX and GFAP (**a**) or Iba1 (**b**) showing the absence of PDDF in an astrocyte and a microglial cell from irradiated rats at 15 days post-IR. Some neuronal nuclei of different size counterstained with DAPI contain γH2AX-positive PDDF. **c** Cerebral cortex cryosection illustrating a ß-galactosidase-positive senescent microglial cell free of γH2AX-positive PDDF at 15 days post-IR. Scale bars: 10 μm

The organization of PDDF was analyzed in dissociated cortical neuron perikarya and cerebral cortex cryosections from rats and mice exposed to a single dose (4 Gy) of IR, known to induce DSBs [7], and examined 15d post-IR. Immunolabeling preparations for the phosphorylated histone H2AX (γΗ2ΑX), a well-established marker of DSBs [19, 33], counterstained with propidium iodide revealed the presence of γΗ2ΑX-positive PDDF in cortical neurons from both species (Fig. 1a-c). PDDF-containing cells were identified as neurons by their large and euchromatic nuclei, prominent nucleoli and distribution of protein synthesis machinery in the Nissl substance (Fig. 1a-c) and further confirmed with immunostaining for the neuronal marker NeuN (Fig. 1d, e). PDDF were found in cortical neurons of different sizes, suggesting that persistent DNA damage occurs in several neuronal types within the cerebral cortex. Moreover, PDDF were not detected in glial cells neither astrocytes nor microglia (GFAP or Iba1 positive, respectively, Fig. 2a, b). It is known that persistent DNA damage induces a senescence-like phenotype in neurons [22]. To explore whether the formation of PDDF was associated with senescence we performed ß-galactosidase immunolabeling in cerebral cortex cryosections. We found very few ß-galactosidase-positive cells, all of them featured by the typical microglial filigrane cytoplasmic processes, and the absence of PDDF (Fig. 2c).

**Fig. 3:**
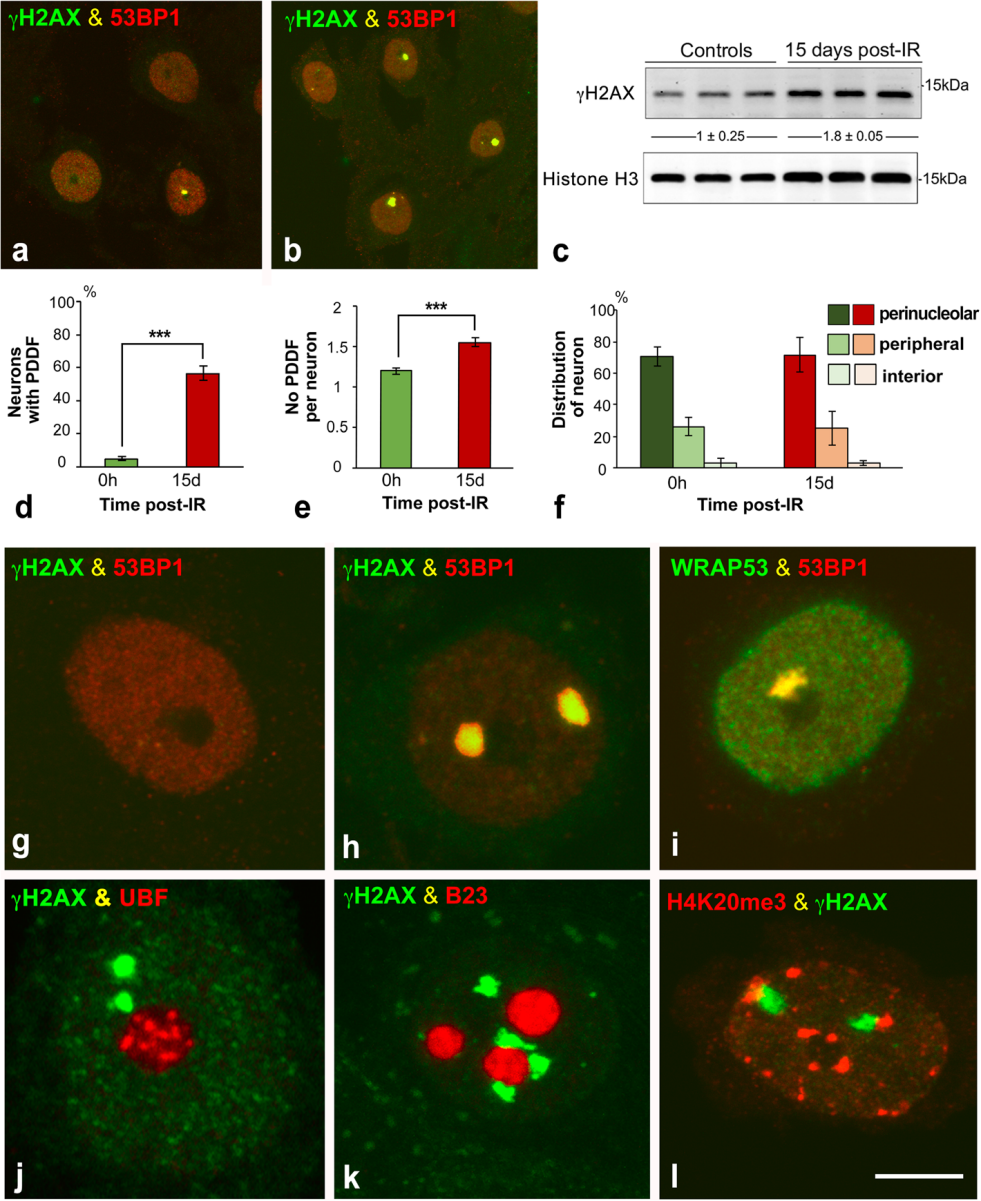
**a, b** Representative examples of double immunolabeling for γH2AX and 53BP1 in dissociated cortical neurons from non-irradiated (**a**) and irradiated rats (15d post-IR) (**b**). Some neurons exhibited a PDDF immunolabeled for γH2AX and 53BP1. Scale bar: 5 μm. **c** Western blot analysis of γH2AX in parietal cortex lysates from non-irradiated and irradiated rats (*n* = 3 animals per group). Protein levels of γH2AX were increased upon DNA-damage induced IR. The expression of histone H3 was used as protein loading control, and the fold increase estimated. **d** Proportion of cortical neurons containing γH2AX-positive PDDF in non-irradiated and irradiated neurons. (***p < 0.001 by Student’s t test). **e** Mean number of PDDF per nucleus within the PDDF-containing neuronal population. (*p < 0.05 by Student’s t test). **f** Distribution of PDDF in three nuclear regions: perinucleolar, nuclear periphery and nuclear interior. Approximately 70% of PDDF were spatially associated with the nucleolus in both non-irradiated and irradiated cortical neurons. **g-l** Double labeling for γH2AX in combination with 53BP1 (**g**, **h**), UBF (**j**), B23 (**k**) or histone H4K20me3 (**l**), and for 53BP1 in combination with WRAP53 (**i**) illustrating the concentration of γH2AX, 53BP1 and WRAP53 in PDDF, and the spatial association of PDDF with the nucleolus (**j**, **k**) and with heterochromatin masses (**l**). g: non-irradiated neuron. **h**-**l**: irradiated neurons at 15 days post-IR. Scale bar: 5 μm

To compare PDDF formation between non-irradiated and irradiated rats, dissociated cortical neurons were immunolabeled for γΗ2ΑX and 53BP1, an essential protein that promotes DNA repair by the NHEJ [4, 60]. 53BP1 showed a general and diffuse nuclear pattern that excludes the nucleolus in control non-irradiated cells (Fig. 3a, g). In control neurons γH2AX- and 53BP1-positive PDDF were infrequent (less than 5%) and likely correspond to spontaneous DNA damage (Fig. 3a, d). In contrast, one or a very few PDDF immunoreactive for γH2AX and 53BP1 were found in about 55% of cortical neurons at 15d post-IR (Fig. 3b, d, h). Among the population of cortical neurons with DNA damage, the mean number of PDDF per nucleus was 1.5 in irradiated neurons and 1.2 in non-irradiated ones (Fig. 3e). In addition to γH2AX and 53BP1, PDDF concentrated WRAP53 (WD40 encoding RNA antisense to p53) (Fig. 3i), an essential protein for DDR that provides a scaffold for DNA repair factors [31]. The persistence of DNA damage was confirmed by Western blotting performed with cerebral cortex lysates, were a significant increase in the expression level of γH2AX was detected at 15d post-IR (Fig. 3c). Our data prove the persistence in PDDF of essential components of the DDR indicating that DNA damage/repair signaling can last in the long term after IR.

Since PDDF nuclear topology affects their possible interactions with other nuclear compartments, we analyzed PDDF distribution in three nuclear domains: perinucleolar, peripheral and nuclear interior. Our results indicate an organized distribution of PDDF within the neuronal nucleus: Approximately 70% of them were perinucleolar, 25% were distributed at the nuclear periphery and the rest were located in the nuclear interior (Fig. 3f). This preferential association of PDDF with the nucleolus was confirmed in cortical neurons immunostained for γH2AX in combination with two nucleolar markers, upstream binding factor (UBF) and nucleophosmin/B23, [32, 58] (Fig. 3j, k). PDDF located at the nuclear periphery or nuclear interior were frequently associated with a heterochromatin mass positive for the histone H4K20me3, a marker of repressed chromatin domains [68], (Fig. 3l).

### PDDF compartmentalization and boundaries in cortical neurons

**Fig. 4:**
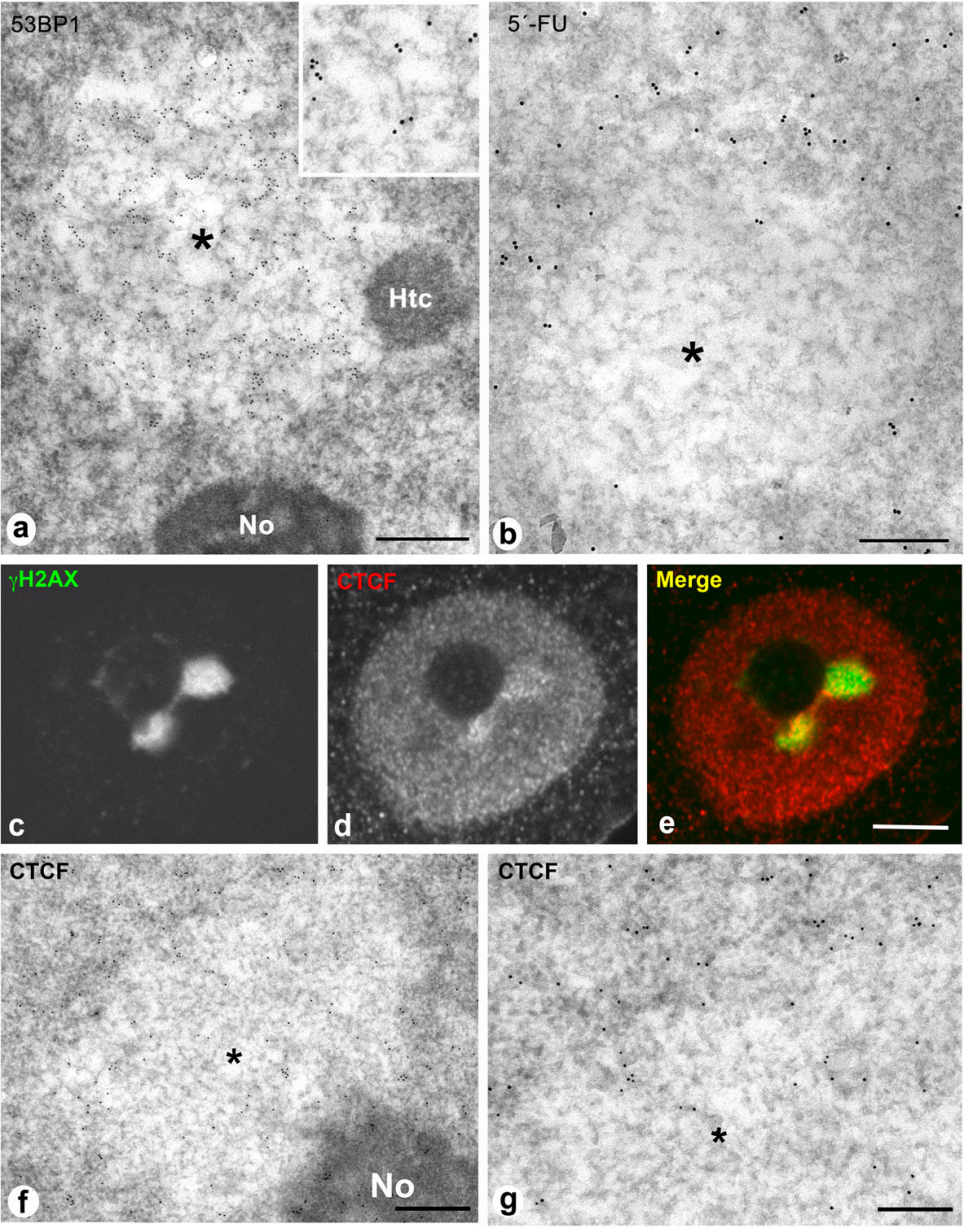
**a** Immunogold electron microscopy for 53BP1 of a typical PDDF (asterisk) in a rat cortical neuron. It is composed of a loosen network of chromatin fibers decorated with gold particles and appears associated with the nucleolus (No) and a heterochromatin mass (Htc). 15 days post-IR. Scale bar: 500 nm Inset: High magnification of 53BP1-immunolabeled chromatin fibers. **b** In situ electron microscopy transcription assay illustrating the incorporation of 5′-fluorouridine (5’-FU) into nascent RNA after 45 min of the administration of the halogenated nucleotide. Note the absence of 5’-FU incorporation in the PDDF (asterisk) and its incorporation in the transcriptionally active adjacent euchromatin. Rat cortical neuron after 15 days post-IR. Scale bar: 300 nm. **c-e** Representative example of double immunolabeling for γH2AX and CTCF in an irradiated cortical neuron showing two perinucleolar PDDF. In addition to a diffuse nuclear distribution of CTCF, this insulator protein appears concentrated in numerous microfoci at the periphery of the γH2AX-positive PDDF. 15 days post-IR. Scale bar: 3 μm. **f, g** Immunogold electron microscopy for the CTCF shows its preferential distribution at the PDDF boundary with euchromatin, although some scattered gold particles appear within the PDDF (asterisk). No: nucleolus. Scale bars: f, 450 nm; g, 250 nm

To define the structural nature of PDDFs we performed immunogold electron microscopy experiments to detect the DNA repair factor 53BP1. PDDF appear as cleared chromatin compartments within euchromatin regions, featured by a loose network of chromatin fibers. Their electro-lucent appearance, decompacted chromatin conformation and well-defined boundaries with adjacent euchromatin, makes the PDDF a distinct nuclear compartment (Fig. 4a). High magnification analysis showed that immunogold particles specifically localize over the network of chromatin fibers within PDDF (Fig. 4a, inset). This observation suggests that the PDDF contain chromatin regions with increased accessibility to DNA-damage repair factors.

PDDF transcriptional activity was determined by means of an in situ transcription assay performed at ultrastructural level. Active transcription was observed in euchromatic domains upon a 45-min pulse of 5’-FU (Fig. 4b). In contrast, PDDF appeared as transcription-free chromatin compartments that lacked nascent RNA. Interestingly, transcriptional activity was observed in their flanking euchromatin, establishing a sharply defined boundary between intact euchromatin, that is transcriptionally permissive, and DNA-damaged chromatin, which is transcriptionally silent (Fig. 4b).

To further understand the molecular organization of PDDF boundaries we performed light and electron microscopy immunocytochemistry to investigate whether CTCF (CCCTC binding factor) is involved in the architectural organization of PDDF. CTCF is an insulator protein that, together with cohesin complex, is responsible for the chromatin folding in Topologically Associating Domains (TADs), submegabase segments that tend to self-associate and define discrete transcriptionally related regions [17, 46, 54]. Our immunocytochemical analysis showed a diffuse pattern of nuclear CTCF binding that is specifically enriched at the borders between PDDFs and the adjacent euchromatin where prominent CTCF-positive microfoci were visible (Fig. 4c-e). Immunogold electron microscopy confirmed CTCF enrichment at the euchromatin flanking PDDF, and a reduced CTCF density inside these regions (Fig. 4f, g). Our findings agree with recently published data supporting that CTCF-dependent chromatin structure is essential to define the chromatin sensitivity to DNA damage [5].

### Genome-wide distribution of γH2AX in IR treated cortical neurons reveals persistent DNA damage in specific genomic regions

**Fig. 5:**
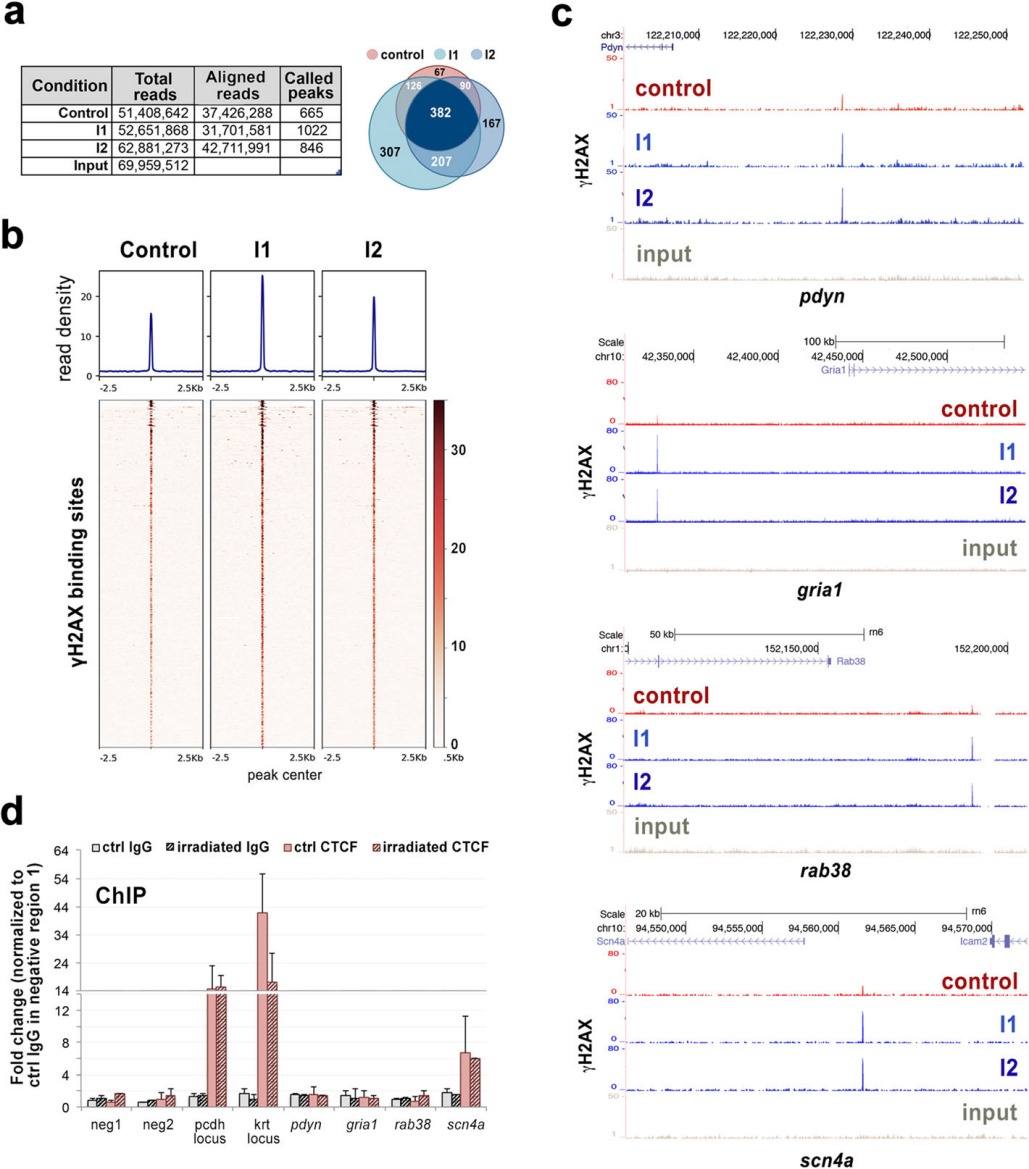
Genome wide distribution of γH2AX in rat cortical neurons 15d upon IR shows persistent DNA damage in specific genomic regions. **a** Table shows the number of total reads, uniquely aligned reads and called peaks for control and irradiated (I1 and I2) rat cortical neurons. Venn diagram shows the overlap (at least one nucleotide) between the called peaks defined in the different conditions. **b** Read density plots and heatmaps show genome-wide γH2AX distribution in the conditions described in **a**. **c** UCSC browser images showing γH2AX binding in different genomic regions close to neuronal specific processes and pathologies. **d** ChIP-qPCR analysis of CTCF enrichment around several γH2AX binding sites defined by ChIP-seq. Immunoprecipitation with rabbit IgG was performed to ensure antibody specificity. Neg1 and neg2 correspond to genomic regions with undetectable γH2AX binding. Pcdh and krt loci were used as positive CTCF binding sites. Graph represents the results of two independent biological replicates performed in triplicates.

We wondered then if persistent DNA damage could be located in genomic regions known to be crucial for neuronal physiology and homeostasis. Since γH2AX is specifically located in PDDFs, we reasoned that γH2AX containing regions would correspond to the part of the genome located in these domains. To address this issue, we performed ChIP-seq analysis in cells from cerebral cortex of control and irradiated rats. In order to be sure about the specificity of the γH2AX binding regions, we performed ChIP-seq in two independent biological replicates of irradiated cells (I1 and I2 in Fig. 5). Moreover, we sequenced about 60 million reads per condition to ensure enough genomic coverage resulting in approximately 30 to 42 million of uniquely aligned reads that defined 665 γH2AX peaks in control cells and a slightly higher number of positions in irradiated cells (1022 and 846) with a FDR < 0.05 (Fig. 5a). The vast majority (90%) of the γH2AX positions found in control cells were also present in irradiated neurons, confirming the existence of genomic regions with higher damage sensitivity (Venn diagrams in Fig. 5a). Importantly, 80% of the γH2AX peaks defined in I2 cells coincided with those in I1 replicate, supporting the robustness and reliability of our analysis. Read density analysis further sustained not only the conservation of γH2AX genomic distribution among conditions, but also the persistency of increased γH2AX levels in cells 15d upon irradiation (Fig. 5b). The analysis of the regions specifically enriched in irradiated cells with *Panther* software revealed that some of them were located at close distance or within the gene body of genes involved in essential functions for neuronal homeostasis, including neurotransmission, synaptic plasticity and adhesion, pentose phosphate pathway, autophagy-lysosomal pathway and protein quality control (Fig. 5c, Additional file 2: Figure S1a and Table 1). In addition, we have identified three genes (*olr551*, *vom1r24* and *vom2r41*) encoding olfactory receptors, which are relevant for olfaction in rodents. The characterization of γH2AX binding sequences suggests that PDDFs contain some cell-specific genes that are normally expressed in neurons. Importantly, 16 of these genes appeared in the OMIM (Online Mendelian Inheritance in Man) catalog of human genes implicated in genetic phenotypes of certain human diseases, most of them neurological and neuropsychiatric diseases (Table 1). They include neurological disorders such as Lafora disease (*epm2a*), encephalopathy familial with neuroserpin inclusion bodies (*serpini1*), mental retardation X-linked 21 (*il1rpl1*) and hyperkalemic periodic paralysis (*scn4a*).

**Table 1:** γH2AX-binding genomic regions specifically enriched in PDDF from cortical neurons and related to human genes implicated in neurological and neuropsychiatric disorders

Gene	Encoded protein	Fold change	Relation to human pathology	Reference
*aga*	Aspartylglucosaminidase	2.875	Aspartylglucosaminuria (0.69)	[34]
*appl2*	Adaptor protein containing Ph domain, Ptb domain and leucine zipper motif 2	3.31	Substance adiction (0.2)	[18]
*cdh10*	Cadherin 10	2.88	Autism (0.2)	[78]
*epm2a*	Laforin glucan phosphatase	2.25	Lafora disease (0.71), progressive myoclonus epilepsy (0.407)	[1, 24]
*fbxo30/fbxw7*	F-box/WD repeat containing protein 7	2.25	Glioma (0.127), genome instability (0.12)	[8, 75]
*gria1*	Glutamate receptor 1	3.72	Schizophrenia (0.208), Bipolar Disorder (0.206), Mental Depression and Depressive disorder (0.201), learning and memory disorders (0.2)	[10, 40, 51, 70]
*gria2*	Glutamate receptor 1	2.69	Schizophrenia (0.209), Bipolar Disorder (0.203), Depressive disorder (0.201)	[9–11]
*htr1a*	Serotonin 5-Ht-1A receptor 1A	2.72	Mental Depression and Depressive disorder (0.253), Schizophrenia (0.221)	[42, 77]
*il1b*	Interleukin 1-β	2.39	Alzheimer disease (0.369)	[80]
*il1rapl1*	Interleukin 1 receptor accessory protein-like 1	2.375	Autism (0.404), Mental retardation (0.205)	[30, 66]
*lbr*	Lamin b receptor	2.39	Reynold syndrome (0.6)	[25]
*rab38*	Ras-related protein Rab-38	3.38	Frontotemporal dementia (0.12)	[20]
*scn4a*	Sodium channel protein type 4 subunit alpha	3.19	Hyperkalemic periodic paralysis (0.688), Potassium-aggravated myotonia (0.602), Hypokalemic periodic paralysis type 2 (0.48), Hypokalemic periodic paralysis type 1 (0.41), Congenital paramyotonia (0.408), Fluctuating myotonia (0.24), Myotonia (0.405)	[6]
*serpini1*	Neuroserpin	2.88	Familial encephalopathy with neuroserpin inclusion bodies (0.681), Dementia (0.208), Progressive myoclonus epilepsy (0.203)	[15]
*sugct*	Succinyl-CoA; Glutarate-CoA transferase	3.71	Migraine (0.24)	[63]
*tkt*	Transketolase	2.23	Wernicke-Korsakoff syndrome, Wernicke encephalopaty (0.201)	[14]

The number in brackets that appears next to the name of diseases corresponds to the “Score of the reliability of the gene-disease pair, based on the type and number of sources where is reported, and the number of PMIDs”

To better define the features of the DNA damage regions found in our analysis we investigated their co-localization with the insulator protein CTCF by ChIP-qPCR. We based on the high degree of conservation between CTCF binding sites among species [71] to infer positive CTCF binding sites in rat cortical neurons. With this aim, we used CTCF and cohesins SMC1 and SA1 ChIP-seq data from adult mouse cortex [16] to select two strong CTCF binding sites (pcdh and krt loci) whose binding region shared more than 80% of identity with the corresponding region in the rat genome (Additional file 2: Figure S1b) and that were used as positive controls for CTCF enrichment. Our ChIP-qPCR analysis revealed that the γH2AX binding site located close to *scn4a* promoter indeed colocalizes with CTCF (Fig. 5d), suggesting that at least a fraction of the DNA damage sensitive sites found in our study might be related to topological restraints.

## Discussion

Increasing evidence supports a role for the accumulation of unrepaired DNA in the ageing process [23, 43, 79] and in the pathogenesis of neurodegenerative disorders [22, 36, 48, 52, 59]. Our study provides the first analysis of the long-term nuclear compartmentalization and genomic localization of unrepaired DNA in rodent healthy cerebral cortex neurons that have been exposed to IR. Here, we found that generation of DSBs in rat and mouse cortical neurons by IR induces de novo formation of a chromatin compartment, the PDDF [7, 50]. We demonstrate that PDDF are neuron-specific structures, as shown by their exclusive presence in cells with the typical neuronal morphology of perikarya [62] that express the neuronal marker NeuN. PDDF delimitate genomic regions in which chromatin conformation cannot be restored to its normal pre-damage state due to persistent DSBs that are difficult to repair or not reparable at the long term [48, 79]. The absence of PDDF in astrocytes and microglia suggests that glial cells are either more resistant to irradiation or more efficient to repair DNA damage. Interestingly, senescence-like state induced by DNA damage [22] seems not to be correlated with PDDF formation upon irradiation, at least under the experimental conditions used in this study.

The organization pattern of PDDF in cortical neurons is similar to that observed in sensory ganglion neurons of the peripheral nervous system under similar experimental conditions [50]. This fact strongly supports that central and peripheral mammalian neurons share a similar pattern of DDR, resulting in the accumulation of unrepaired DNA in a specific nuclear compartment, the PDDF.

The presence of PDDFs in most cortical neurons at 15d post-IR indicates that unrepaired DNA sequences from different chromosomes, as revealed the ChIP-seq analysis, move from over relatively large distances to be concentrated in one or two isolated chromatin compartments. Increased chromatin mobility at sites of IR-induced DSBs has been previously reported by tracking the fluorescently tagged DNA repair factor 53BP1 in living mammalian cells [38]. In line with this, recent work has revealed that 53BP1 promotes the mobility of damaged chromatin [81].

PDDF appeared as cleared chromatin domains with a decompacted structure composed of loosely organized chromatin fibers [39, 50]. This configuration likely provides DNA repair factors a better access to damaged DNA, as suggested by the 53BP1 immunogold labeling of chromatin fibers within PDDF. Interestingly, although PDDF exhibit an open chromatin structure, which is in principle permissive to gene expression, they are transcription-free nuclear compartments. Transcriptional silencing at PDDF could thus be a protective neuronal mechanism aimed to reduce genomic instability specifically in neurons by preventing the production of aberrant mRNAs and proteins encoded by damaged genes [50]. It is important to consider that neurons rapidly repair most DNA lesions within 24 h post-IR to promote cell survival [7] and that the NHEJ DNA repair pathway is error-prone and occasionally works at the expense of small deletions and mutations that can provoke transcriptional errors [13, 27, 79]. Thus, in spite of the protective role of PDDF, those transcriptional errors can lead to neuronal dysfunction by affecting the cellular proteostasis [27].

An important challenge is to understand how neurons tolerate DNA damage accumulation without triggering neurodegeneration and cell death in spite of the numerous DSBs induced upon a single dose of IR [7, 50]. Our results suggest that PDDF are specialized nuclear centers for long-term sequestration of unrepaired DNA, which maintain the neuronal DNA damage/repair signaling (γH2AX and 53BP1) and prevent the expression of damaged genes. By sequestering damaged DNA, PDDF would help protecting genomic integrity and avoid transcription of undamaged chromatin, therefore contributing to neuronal survival. Since mammalian neurons are diploid cells [61], the transcriptional blockade of the genes located in the genomic regions contained within the PDDF could potentially be compensated by the expression of the second copy of the gene. In fact, our in situ transcription assay reveals that transcription is preserved in undamaged euchromatin, including the flanks of PDDF.

One important issue is to understand how the specific structural, molecular and transcriptional features of the PDDF, delimited by their well-defined boundaries, are established. Genome-wide interaction studies by chromosome conformation capture techniques have shown that the genome is organized in Topologically Associated Domains (TADs) that constitute discrete regulatory units within which enhancers and promoters interact [17, 55]. TADs are separated by boundary regions that often have cohesin and CTCF [17]. Disruption of CTCF binding sites by CRISP/Cas9 genome editing impairs the insulation activity of TAD boundaries and provokes changes in the enhancer-promoter interaction profile that leads to changes in transcription [28, 46]. Our findings showing CTCF enrichment at PDDF borders as well as its colocalization with the γH2AX binding site defined upstream the *scn4a* gene points to a role of CTCF, likely in cooperation with cohesin complex, in the definition of the interface between healthy and damaged chromatin. In agreement with this, it has been recently published that chromatin loop anchors bound by CTCF and cohesin are specially vulnerable to DSBs induced by topoisomerase 2B [5]. These data indicate that there is a narrow relationship between chromatin architecture and topological stress. Moreover, the insulation function of CTCF at the PDDF boundaries might be necessary for transcriptional repression and active clustering of damaged DNA sequences. In cortical neurons, this mechanism could contribute to tolerate the accumulation of unrepaired DNA in an insulated compartment without triggering apoptotic pathways. This idea is supported by experiments performed in a different cellular model, where CTCF boundaries located in repressive heterochromatin domains separate enhancers from promoters thus silencing transcription [64].

PDDF are not randomly distributed inside the nuclear volume of cortical neurons; rather than that, they have a preferential spatial association with the nucleolus and, to a lower extent, with the heterochromatin masses located at the nuclear periphery. In both localizations PDDF are closely associated to a repressive nuclear environment [64]. In mammalian neurons, such repressive environment is specifically generated by the clustering of centromeric and telomeric heterochromatin domains at the nucleolar surface and nuclear periphery [2, 49]. It is well established that repressed heterochromatin is enriched in silencing proteins such as the methylated DNA-binding protein MeCP2 and polycomb repressive complexes (PRC1 and PRC2) whose presence at those positions can silence nearby genes [2, 64]. Localization of PDDF adjacent to a repressive environment might facilitate the selective silencing of damaged genes, as revealed by the in situ transcription assay, thus contributing to preserve genomic stability. Moreover, several lines of evidence indicate that nucleolar and heterochromatin DSBs can move towards the periphery of both structures for DNA repair looking for the more permissive environment constituted by euchromatin [12, 29, 35, 76]. This raises the possibility that PDDFs could also be involved in DNA repair of ribosomal genes and heterochromatin sequences.

A major finding of this study is the genome-wide identification of DNA sequences enriched in PDDF from cortical neurons. Given the specific binding of γH2AX to damaged chromatin within PDDF, we assume that our ChIP-seq signal corresponds to genomic regions located inside this nuclear compartment. To our knowledge this is the first study characterizing the genomic distribution of neuronal unrepaired DNA accumulated in nuclear foci. Importantly, our analysis revealed that γH2AX was already present in certain genomic positions in non-irradiated neurons and that upon irradiation, its levels increase to a different extent depending on the regions, ranging from 1.5 to 7.5 fold. This observation is consistent with our results in cortical neurons (present study) and previous data from sensory ganglion neurons [50] that show that approximately 5% of non-irradiated neurons exhibit PDDFs, indicating that young adult neurons can accumulate unrepaired DNA for long-term under physiological conditions. Based on this, we envision that there could be a set of genes which, by virtue of their genomic distribution in chromatin domains, are more vulnerable to DNA damage or more refractory to DNA repair and whose unrepaired DNA accumulates in PDDFs. This hypothesis is supported by their increased vulnerability to IR exposure, as shown by the higher levels of γH2AX observed in irradiated cerebral cortex samples.

It has been proposed that neurons, as post-mitotic cells, avoid repairing their whole genome and limit to repair specifically a few genes through a mechanism that is coupled to their transcription [56, 57]. Transcription of these genes is important for maintenance of trophic functions required for neuronal survival. However, our results indicate that most of γH2AX located close to protein-coding genes correspond to cellular pathways essential for neuronal homeostasis, such as neurotransmission, synaptic plasticity and adhesion, pentose phosphate pathway or autophagy-lysosomal pathway. This suggests that at least a set of actively transcribed genes could be inefficiently repaired, and remain at PDDFs.

In addition to ageing, defective DNA repair and concomitant DNA damage accumulation has been linked with several neurodegenerative disorders [13, 48, 52, 73]. However, understanding the pathogenic mechanisms of DNA damage requires the identification of those genes with higher propensity to accumulate lesions in neuropathological conditions. A major challenge is to identify the DNA lesions essential for the progression of each neurodegenerative disease by using experimental animal models in which the accumulation of DNA damage mimics partially the pathogenesis of the human disease [48]. In this regard, our γH2AX ChIP-seq analysis identifies a number of genes with nearby DNA lesions in both non-irradiated and irradiated samples, suggesting that they are more susceptible to be damaged. Some of these genes are cataloged in OMIM and DisGeNET platforms as associated with genetic human diseases, with special focus in neurological and neuropsychiatric disorders. Among those related with neurological disorders, *epm2a* (Lafora disease), *serpini1* (encephalopathy familial with neuroserpin inclusion bodies), *il1rpl1* (autism and mental retardation X-linked 21) and *scn4a* (hyperkalemic peridoc paralysis), are particularly interesting for their essential role in these diseases, indicated by the “reliability score” of the gene-disease pair (Table 1). Based on this, we believe that our results open a promising possibility to design proper genetically modified animal models of neurological disorders aimed to investigate the molecular mechanisms that prevent the repair of vulnerable genes.

## Additional files

Additional file 1: **Table S1.** Primers used for ChIP-qPCR in different rat genomic regions. (XLSX 62 kb)

Additional file 2: **Figure S1.** **a** UCSC browser images showing γH2AX binding around *appl2* gene. **b** UCSC browser images corresponding to the mouse pcdh and krt loci located in chromosomes 11 and 18 respectively and their corresponding regions in the rat genome. Binding sites defined by ChIP-seq for cohesin subunits SMC1 and SA1, as well as for CTCF in mouse adult cortex are shown. Arrowheads show the position of the primers used for ChIP-qPCR performed with chromatin from rat cortical neurons. (JPG 1740 kb)

## Abbreviations

ChIP: Chromatin immunoprecipitation
DDR: DNA damage response
DSBs: Double Strand breaks
IR: Ionizing radiation
NHEJ: Non-homologous end joining
OMIM: Online Mendelian Inheritance in Man
PDDF: Persistent DNA damage foci
TADs: Topologically Associated Domains
