# Supplementary figures and images for: Persistent accumulation of unrepaired DNA damage in rat cortical neurons: nuclear organization and ChIP-seq analysis of damaged DNA
Source: Acta Neuropathol Commun. 2018 Jul 26;6:68. doi: 10.1186/s40478-018-0573-6 (PMC6062993; doi:10.1186/s40478-018-0573-6)

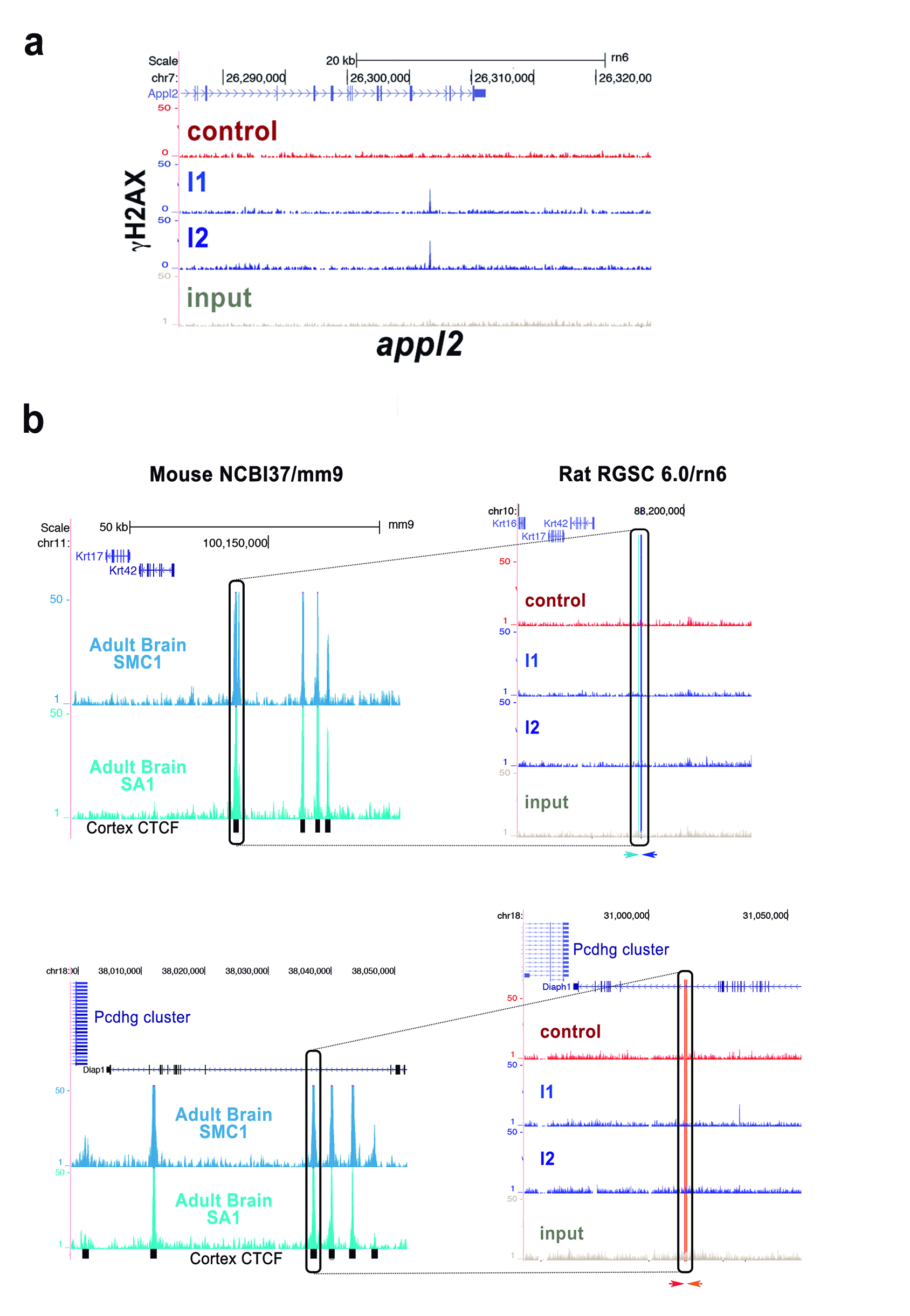

Supplement: Supplementary file 2 — Figure S1. a UCSC browser images showing γH2AX binding around appl2 gene. b UCSC browser images corresponding to the mouse pcdh and krt loci located in chromosomes 11 and 18 respectively and their corresponding regions in the rat genome. Binding sites defined by ChIP-seq for cohesin subunits SMC1 and SA1, as well as for CTCF in mouse adult cortex are shown. Arrowheads show the position of the primers used for ChIP-qPCR performed with chromatin from rat cortical neurons. (JPG 1740 kb) [file 40478_2018_573_MOESM2_ESM.jpg]
